# Understanding evidence ecosystems: What influences the production, translation, and use of modeled evidence in Burkina Faso, Nigeria, India, and Kenya?

**DOI:** 10.12688/gatesopenres.14973.1

**Published:** 2023-12-13

**Authors:** Ali Sié, Habibou Fofana, Moubassira Kagoné, Moussa Ouédraogo, Obinna E. Onwujekwe, Chinyere O. Mbachu, Maulik Chokshi, Latha Chilgod, Tushar Mokashi, Arun B. Nair, Peter Muriuki, Abeba Taddese, Leah Ewald, Apoorva Handigol

**Affiliations:** 1 Centre de recherche en santé de Nouna, Ouagadougou, Burkina Faso; 2 Health Policy Research Group, University of Nigeria, Enugu, Nigeria; 3 ACCESS Health International, New Delhi, India; 4 Independent, Nairobi, Kenya; 5 Results for Development Institute, Washington, District of Columbia, USA

**Keywords:** Evidence use, modeling, evidence ecoystem, public health disease modeling, public health, disease modeling, boundary organization, knowledge broker, evidence to policy

## Abstract

**Background:**

This study sought to document and understand facilitators and barriers to producing, translating, and using modeled evidence in decision-making in Burkina Faso, Nigeria, India, and Kenya. We explored researcher-decision-maker engagement mechanisms as key facilitators of evidence use, with a focus on knowledge brokers and boundary organizations.

**Methods:**

The study used mixed methods drawing on analysis from key informant interviews and surveys, complemented by a rapid desk review to map modeling activities and actors. The survey was conducted online while the qualitative research entailed in-depth interviews with modelers, knowledge brokers, and decision-makers working in a representative variety of health fields, organizations, and levels of government. This study was approved by Health Media Lab IRB (Institutional Review Board) in the United States and a local IRB in each study country and conducted between September 2021 and June 2022.

**Results:**

Informants interviewed for this study described a range of factors that facilitate and inhibit the use of modeled evidence in public health decision-making at the individual, organizational, and environmental levels. Key themes included the capacity to produce, translate, and use modeled evidence; the timing and relevance of modeling outputs; the existence of communications channels between modelers and decision-makers; the strength of underlying data systems; the role of sustained funding; and the impact of global crises.

**Conclusion:**

This study highlights the importance of taking an ecosystem approach to supporting modeling activities, considering individual, organizational, and environmental factors and how different actors and interact to inform the production, translation, and use of modeled evidence. Structured interaction that promotes dialogue, debate, and joint sense making between the producers and users of evidence is critical to informing and influencing the use of evidence in decision-making.

## Introduction

Insufficient use of evidence in public health decision-making, including modeled outputs, can result in losses in efficiency, effectiveness, and impact that affect the end users of a health system. The gap between the production of evidence and its use in policy and practice is commonly attributed to barriers that include limited access to relevant research, misaligned time frames, lack of policymaker skills, and limited research capabilities ( Campbell *et al.*, 2009; Oliver *et al.*, 2014). Engagement mechanisms that are designed to bring researchers and decision-makers together to foster co-creation, knowledge sharing, debate, and dialogue are a way to bridge this gap and facilitate the use of evidence ( Campbell *et al.*, 2009; Oliver *et al.*, 2014; Robert *et al.*, 2020; Taddese, 2021).

Different terms are used in the literature to describe how this type of collaboration and exchange can be facilitated, including using knowledge brokers, intermediaries, boundary organizations, and knowledge translators ( Cvitanovic, 2018; Taddese, 2021). While precise definitions can help us better understand and distinguish the contributions of each of these approaches ( Neal *et al.*, n.d.) in facilitating the use of evidence, these terms are often used interchangeably. Importantly, however, they call attention to the need for deliberate spaces or mechanisms to bring evidence to policy and practice ( Hinrichs-Krapels *et al.*, 2020; Issues, 2022).

The coronavirus disease 2019 (COVID-19) pandemic put a spotlight on the role public health disease modeling can play in helping to guide policy and planning decisions. It also illuminated the complexities and challenges associated with communicating modeled results. Decision-makers are reluctant to communicate findings they do not understand and modelers face difficulties in rapidly responding to decision-maker demands and communicating complex models and outputs in easy-to-understand formats (GAO-20-372, Infectious Disease Modeling). Mechanisms designed to intentionally bring modelers and decision-makers together help to build trust – providing a space in which decision-makers can work with modelers to refine questions and gain improved awareness of the value of using modeled results ( Hinrichs-Krapels *et al.*, 2020; Ward *et al.*, 2009; Williamson *et al.*, 2019). Through this engagement, researchers, in turn, can develop a better understanding of decision needs and how to tailor complex results and messages ( Hinrichs-Krapels *et al.*, 2020; Ward *et al.*, 2009). To date there has been little documentation of engagement mechanisms specific to disease modeling.

To address this gap in knowledge, we conducted a mixed methods study in four countries – Burkina Faso, Nigeria, India, and Kenya -- between September 2021 and June 2022. A key objective of our research was to document and understand facilitators and barriers to producing, translating, and using modeled evidence in decision-making. We explored researcher-decision-maker engagement mechanisms as key facilitators of evidence use, with a focus on knowledge brokers and boundary organizations. Specifically, we were interested in the following research questions:

1. What factors facilitate or inhibit exchange between decision-makers and modelers?
2. What do partnership structures that support exchange between decision-makers and modelers look like in different contexts? How do they work?
3. How can funding mechanisms, organizational structures, and other practices be improved to better support partnership structures that facilitate exchange between decision-makers and modelers?

## Methods

### Ethics approval

This study was approved by Health Media Lab IRB in the United States (HML IRB Review ID: 1001R4DV21, October 8, 2021) and a local IRB in each study country (Comité d’ethique pour la recherche de santé in Burkina Faso, Health Systems Research India Initiative Institutional Ethics Committee in India, Amref Ethics and Scientific Review Committee in Kenya, and the University of Nigeria Teaching Hospital Health Research Ethics Committee in Nigeria). In Kenya, the study was also registered with the National Commission for Science, Technology and Innovation, per local regulations (Ref No. 801251, December 1, 2021).

### Study design

The study was coordinated by Results for Development in the United States and funded by the Bill & Melinda Gates Foundation (BMGF).

**Figure 1.**
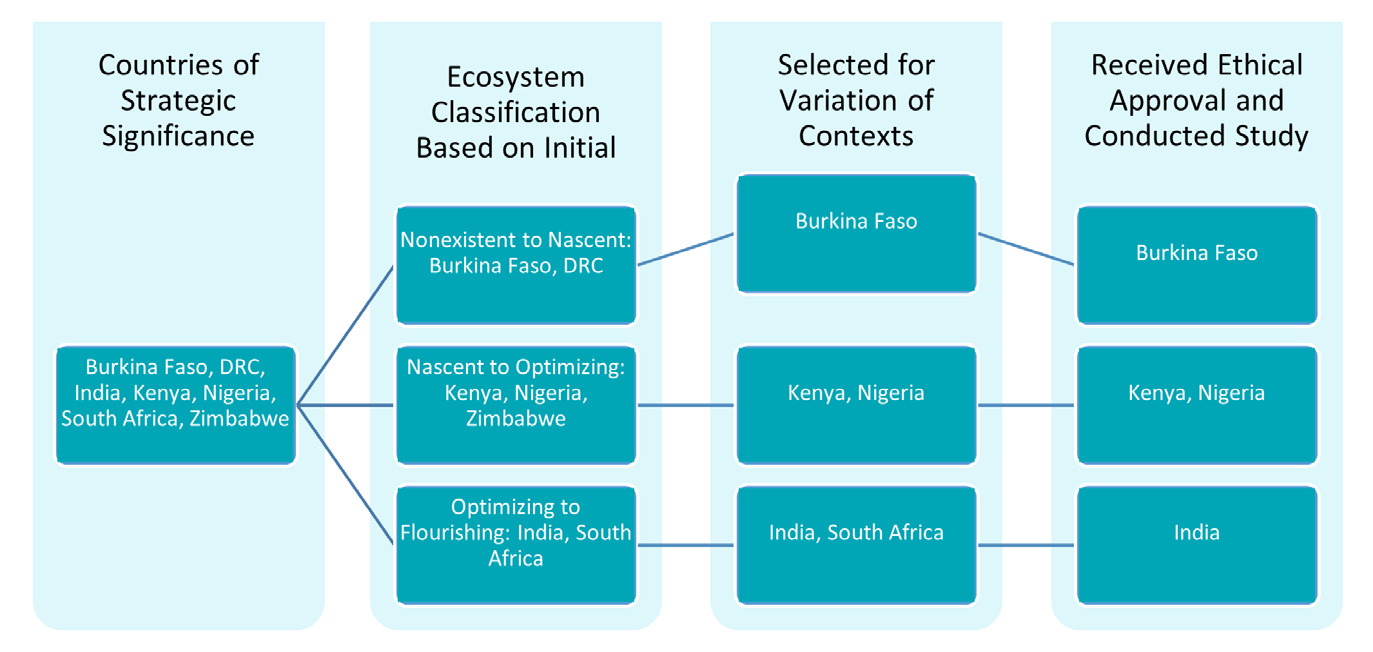
Country Selection Process.

Before country selection, Results for Development conducted a desk review of modeling activities and actors in seven countries considered of strategic importance to BMGF. In combination with informal conversations with key stakeholders, the study team used this desk review to classify each country by level of modeling ecosystem maturity, ranging from “nonexistent” to “flourishing” ( Figure 1). Four countries were then selected to include a variety of maturity levels, geographic regions, and both Francophone and Anglophone representation: Burkina Faso, India, Nigeria, and South Africa. Kenya was added to this list, with a smaller scope and budget, at BMGF’s request to inform planned activities. South Africa was later removed from the study due to delayed ethical review timelines during the COVID-19 pandemic.

Data collection and analysis were carried out by local research partners in the four selected countries: Centre de recherche en santé de Nouna in Burkina Faso, the Health Policy Research Group University of Nigeria in Nigeria, ACCESS Health International in India, and independent consultant Peter Muriuki in Kenya. The research teams applied a mixed-methods approach including a survey and in-depth key informant interviews with 230 decision-makers, modelers, and brokers of modeled evidence across each country’s modeling-to-decision-making ecosystem.

The study was advised by a Working Group that included representatives of stakeholder groups in the four final research countries and South Africa. It met four times during the study. Members were selected by the country research teams and played key roles in the modeling-to-decision-making ecosystem in their country. The mandate of the Working Group was to:

- Root the research in country contexts and priorities.
- Provide a platform for country actors to learn from the experiences of other members.
- Share feedback with donors and other global partners about good practices for enhancing access to and use of high-quality modeled evidence for country-level decision-making.

### Data collection

Results for Development drafted the research tools, including an interview guide and survey. These tools were reviewed by the Global Center for Gender Equality at Stanford University to ensure gender-related issues were taken into consideration and then lightly adapted by the country teams to reflect local contexts, address local IRB requirements, and (in Burkina Faso) translate to French. The interview guide and survey can be found as *Extended data* ( Taddese *et al.*, 2023).

In Burkina Faso, Nigeria, and India, the country research partners generated initial lists of contacts to receive the survey based on their own and the Bill & Melinda Gates Foundations’ knowledge of who was currently active in the modeling space. Snowball sampling was used to expand this list to further stakeholders. The survey was conducted online and included a question about whether the respondents were willing to be contacted for an interview. In Kenya, the team did not conduct a survey due to the study’s more limited scope and budget.

The research teams identified key informants for interviews from the pool of survey respondents who had provided informative survey responses or through additional outreach efforts to key stakeholders in the modeling space who had not responded to the survey. The research teams aimed to interview modelers, knowledge brokers, and decision-makers working in a representative variety of health fields, organizations, and levels of government, and to ensure inclusion of respondents who identified as women. Further snowball sampling was used to identify additional interview respondents from the actors mentioned by key informants. In Kenya, key informants were identified based on the researcher’s own and the Bill & Melinda Gates Foundations’ knowledge of who was active in the modeling space, along with snowball sampling.

Key informant interviews were conducted in-person or remotely via a virtual platform such as Zoom and were audio recorded. The interviews were conducted by one or two members of the research team in each country, on average taking 30–60 minutes.

The research teams in Burkina Faso, Nigeria, and India continued interviewing until they felt they had reached saturation, at 24 or 25 interviews. The researcher in Kenya conducted nine interviews due to the more limited scope and budget. All the Kenya respondents identified as modelers or decision-makers, with none having a purely knowledge brokering role. The table below summarizes the number of survey respondents and key informants in each country and the survey response rate ( Table 1).

**Table 1.** Survey respondents and key informant interviewees in study countries.

Country	Survey Respondents				Survey Response Rate	Key Informant Interviewees			
	Modeler	Knowledge Broker	Decision- Maker	Total		Modeler	Knowledge Broker	Decision- Maker	Total
**Burkina** **Faso**	20	19	15	54	74.0% (54/73)	7	11	7	25
**India**	10	32	13	55	59.1% (55/93)	6	12	7	25
Kenya*	-	-	-	-	-	4	0	5	9
**Nigeria**	14	7	17	38	52.8% (38/72)	6	4	14	24
**Total**	**44**	**58**	**45**	**147**	**61.8%** **(147/238)**	**23**	**27**	**33**	**83**

### Data analysis

The research teams conducted a quantitative analysis of survey results in Excel using a template provided by Results for Development.

Results for Development created a uniform codebook in Excel to be used to code key informant interviews across all countries. The codes were thematic and broadly grouped by research question. Each research team then had the chance to develop subcodes specific to their context and the data they collected. Each of the research teams coded their first two interviews with a member of the R4D team and held a meeting to discuss their coding, any questions that came up, and ensure uniformity of analysis across countries. The remaining interviews were then coded by one or two members of each research team.

The research teams presented their survey and key informant interview findings to the Working Group to receive feedback and further insights. Each research team then developed a country case study in PowerPoint and policy brief in Word. These analyses fed into a final project report produced by Results for Development summarizing findings across all countries.

This study has a few limitations. First, as the research teams relied on their knowledge and networks to recruit participants into the study, they may have missed other key individuals in the modeling to decision-making ecosystem. Second, survey response rates were low, particularly the response rates from women were low. ^1^ Third, the research teams were unable to interview several of the key informants who participated in the survey and missed the opportunity to build on and further explore their responses. Finally, we found little documentation of country-level public health modeling activities in the study countries. As such, along with the limited scope and condensed timeline of our research, we are likely to have missed some key actors and activities in the modeling ecosystems of the four study countries.

## Results

Overall, 22% of survey respondents (20% in Burkina Faso, 21% in Nigeria, and 24% in India) and 22% of key informants (24% in Burkina Faso, 17% in Nigeria, 20% in India, and 33% in Kenya) identified as women. The rest identified as men. Overall, 20% of survey respondents (7% in Burkina Faso, 26% in Nigeria, and 29% in India) and 25% of key informants (16% in Burkina Faso, 13% in Nigeria, 56% in India, and 0% in Kenya) came from subnational levels. The rest came from the national level, including international organizations working in the country.

### Modeling to policy ecosystem

**Figure 2.**
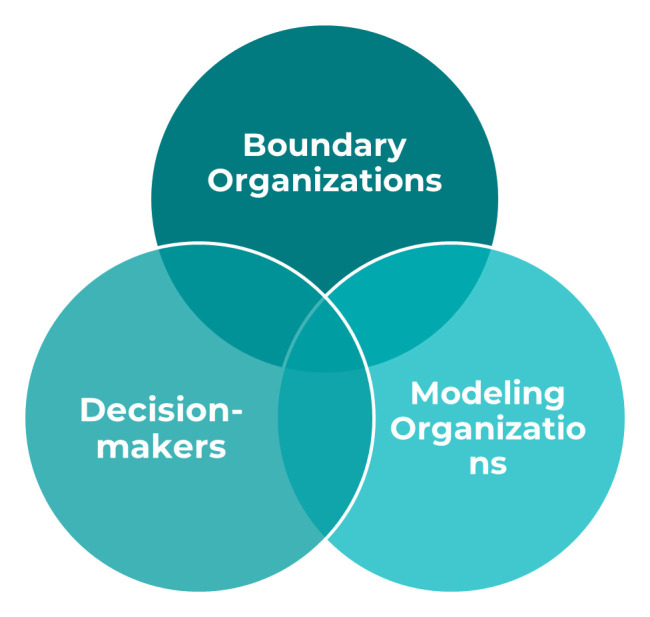
Key Actors in the Modeling to Decision-making Process.

Key actors in the modeling-to-policy ecosystem include modelers, knowledge brokers and boundary organizations, mechanisms or spaces for knowledge sharing and exchange, and funders. Figure 2 and Table 2 identify these actors and provide the definitions we used in the study). Although these terms, particularly boundary organization, knowledge broker, and translator are often used interchangeably in the literature, the distinctions offered in our definitions allowed us to better characterize and describe how modelers and decision-makers come together to inform policy and practice or program level decisions. We observed each of these actors in the four study countries with variation in prominence and role played.

**Table 2.** Definitions Used in this Study.

**Modeling organizations**	In-country or international organizations/researchers that **produce modeled evidence**
**Boundary organizations**	**Stand-alone organizations** that help to distill findings and present them in easy-to-understand formats, foster dialogue, and exchange, and engage decision-makers and modelers in debating the impact of evidence on policy or practice
**Knowledge-brokers**	Individuals or entities typically **embedded within research / modeling organizations** that help to distill findings and present them in easy-to-understand formats, foster dialogue, and exchange, and engage decision-makers and modelers in debating the impact of evidence on policy or practice
**Knowledge translation** **or translation**	The process of **putting evidence into a format that is easy for decision-makers to understand and** **use**
**Decision-makers**	Users/potential users of modeled evidence and those who **participate in making decisions** for national and-subnational health policies and strategies

***Modelers***

**Public universities and research institutions (national and international) play a lead role in developing models in the four study countries.**

In **Burkina Faso**, modeling is conducted almost exclusively by national research and academic institutions.

In **Nigeria**, modeling is conducted primarily by public universities and private academic institutions (local and international) as well as some parastatal research institutions and local NGOs (Non-Governmental Organizations) like Pro-Health Nigeria.

In **India**, a range of actors, including local public and private research and academic institutions, local and international NGOs, government think tanks, and the Health Technology Assessment agency engage in modeling.

In **Kenya**, local universities are the most prominent modelers. But a range of other actors, including the parastatal Kenya Medical Research Institute, bilateral agencies, foreign universities, and regional initiatives, also engage in modeling activities.

***Knowledge brokers and boundary organizations***

The extent to which research organizations with modeling capacity or stand-alone organizations with boundary spanning roles engaged in knowledge brokering to influence policy or practice varied by country. In countries where we observed more modeling activities, we noted an overlap in modeling and knowledge brokering functions, with most research organizations playing both roles.

In **Burkina Faso**, government agencies, civil society organizations, iNGOs, and UN agencies serve as boundary organizations – facilitating communication between modelers and decision-makers without conducting any modeling themselves. Communication between modelers who are not directly affiliated with the government and government agencies is facilitated almost exclusively by these boundary organizations.

In **Nigeria**, the Academy of Science serves as a prominent boundary organization, as well as some local NGOs and UN/bilateral agencies. However, other local NGOs, parastatal organizations, and UN/bilateral agencies both create models and communicate the results directly to the government.

In **India** and **Kenya**, all the organizations that engage in knowledge brokering between modelers and decision-makers are engaged in modeling themselves, including local and international NGOs, government think tanks, parastatal research and academic institutions, and the Health Technology Assessment agency.

***Mechanisms of knowledge exchange***

Across all research countries, government-led advisory groups, working groups, and task forces were routinely cited as key mechanisms for knowledge exchange between modeled evidence and policymaking. Non-government research coalitions also provide a platform for debate and knowledge dissemination, particularly in the countries we observed to have large and complex data ecosystems.

In **Burkina Faso**, government advisory groups, such as the COVID-19 thematic working group, provide a platform where modelers and decision-makers can come together to discuss modeled evidence around various diseases, most prominently COVID-19.

In **Nigeria**, disease-specific and general health or data government advisory groups provide this platform, along with the prominent and independent National Council on Health.

In **India**, independent consortia of researchers such as the COVID-19 consortium and Health Technology Assessment (HTA) consortium provide this platform, while government-led “working trainings” provide unique opportunities for modelers to come together to develop models needed by decision-makers while developing their own modeling capacities.

In **Kenya**, disease-specific government advisory groups and task forces (most prominently the COVID-19 Task Force) provide this platform, while formal partnerships between government and modeling agencies provide another avenue for communication.

In the four study countries, decision-makers currently engaging with modeled outputs reported a focus on disease-specific initiatives, particularly HIV, tuberculosis, and malaria. In all the countries, modelers from all fields were called on to support COVID-19 modeling. Table 3 presents a list of initiatives for which there were past or present modeling efforts in the study countries, as mentioned by key informants. It is not a comprehensive list of all modeling efforts in the research countries.

**Table 3.** Focus of modeling activities across study countries.

	HIV	TB	Malaria	Dengue	NTDs	Rabies	Influenza	COVID-19
**Burkina Faso**	**✓**	**✓**	**✓**	**✓**				**✓**
**India**	**✓**	**✓**	**✓**					**✓**
**Kenya**		**✓**	**✓**		**✓**	**✓**	**✓**	**✓**
**Nigeria**	**✓**	**✓**	**✓**					**✓**

### Facilitators & inhibitors influencing production, translation, and use of modeled evidence

Our research examines the factors that facilitate and inhibit the production, translation, and use of modeled evidence at the interpersonal, organizational, and environmental levels ( Table 4). Interpersonal factors explore capabilities at an individual level, while organizational factors describe the structures, communication, and coordination infrastructure needed to support production, translation, and use of modeled evidence. Finally, environmental factors refer to internal and external conditions that influence the use of evidence, including funding and global events like the COVID-19 pandemic.

**Table 4.** Summary of Interpersonal, Organizational, and Environmental Factors Influencing Production, Translation, and Use of Modeled Evidence.

	Production of modelled evidence	Translation of modelled evidence	Use of modelled evidence
**Interpersonal factors**	Credible capacity		
	Tools and infrastructure for modelling	Understanding of modeling and value of modeled outputs	
**Organizational factors**		Direct access to high level decision makers	Misaligned time frames for modelling and decision-making
	Software and databases for secure transmission of data (information sharing)		Competing time demands
**Environmental factors**	Access to quality data		Context-appropriate models
	Sustained funding for modelling		Emerging diseases (such as COVID-19)

***Interpersonal factors***

Key informants highlighted the importance of having credible modeling capacity across different disciplines to inform policy and practice decisions. Building this type of capacity is time and resource intensive. In addition to individual level capabilities, successful modeling efforts also demand tools and infrastructure for modeling, including modeling software and computers powerful enough to run it.

*“If you take health services, for example, there are very few people who can really look at the data analysis, and that kind of capacity building doesn't happen…nowadays it's an age of data we need more and more people who can look at data and build models and draw conclusions and advise the policymakers. So, at several levels, we need capacity building both in generating data as well as in what I would call crunching data.”*
- Knowledge Broker, India
*“The other problem we have is sometimes the lack of software. Often there are models that you want to make, but it requires the use of particular software that you do not have.”*
- Modeler, Burkina Faso

Modeling outputs can be complex and hard for decision-makers to make sense of and use in informing policy and planning. As a result, they may not understand the value and relevance of the models in addressing the real-life public health questions they are grappling with. Boundary organization representatives reported that when they engage with a decision-maker who understands modeling as a concept, the decision-maker is more likely to promote and accept the usefulness of the model.

*“So just ensuring that we continue to capacity build to understand our data, so that when you are documenting right from the source, and you are able to consume that data.”*
-Decision-Maker, Kenya

Informants also noted that when decision-makers and modelers collaborate in defining the research and policy questions and have regular and sustained engagement with each other, modeled outputs are more likely to be considered in decision making. In this collaboration, it is equally important that modelers communicate and share findings in formats that are easy for decision-makers to understand and use.

*“Getting [decision-makers’] trust and confidence in the first instance in the model output is often what one needs to overcome. Fortunately, interpreting the models in a very clear way that will enable them to see through empirical evidence of what is happening in the sector proves profoundly successful.”*
-Knowledge Broker, Nigeria
*“So, the only time that your research findings can end up with the decision-makers, you must deliberately engage the Ministry from the beginning, you set up stakeholders’ meeting, you develop a policy brief, so I can tell you that it is not a walk in the park.”*
-Modeler, Kenya

***Organizational factors***

Misaligned time frames for modeling and decision-making were a frequently cited issue, particularly in the context of a health emergency like COVID-19. In such situations, decision-makers demand rapid responses to their evidence needs, but high-quality, rigorous models take time to develop. These misaligned expectations can result in modeling outputs only being published once the window for decision-making has already passed.

*“So, you can do a methodology workshop where you look for ideas from them [decision-makers]. At the end of the study, you also go back to them to do dissemination workshop and get their feedback. That kind of arrangement is time consuming.”*
-Modeler, Nigeria
*“So, if…the modeled data is availed on time, then it would enable…a better response.” *
-Decision-Maker, Kenya
*“The study was commissioned but it took too long. Actually, the conduct of HTA (Health Technology Assessment) took I think more than a year's time, and by then… the decision already was taken by the, it was Maternal Child Health Division of the Ministry.”*
-Modeler, India

Decision-makers often report not having enough time to engage in knowledge exchange activities with researchers due to competing time demands. When consulted and engaged in developing models, however, they are more likely to buy in to the process and outputs and consider them in a decision process. Co-production is a way to build decision-maker trust and increase awareness and understanding of the value of using modeled outputs to inform policy and planning. In addition to formal partnerships, informal relationship-building activities can help to position researchers as trusted experts or partners for decision-makers.

*“…In terms of engagement, having the government engaged at whatever stage but engaged in a much more not just, 'I am talking to you and I'm telling you what to do' kind of way, but in a much more ownership kind of a way, always helps.”*
-Knowledge Broker, India
*“Getting their trust and confidence in the first instance in the model output is often what one needs to overcome. Interpreting the models in a very clear way will enable them to see through empirical evidence of what is happening in the sector or another. The approach [we used] really was to co-produce models with the actors…every Tuesday evening, the modelers were meeting with policymakers and the programmatic people. I think that was definitely one strong strategy.”*
-Decision-Maker, Nigeria
*The other one is to involve the protocol development from the beginning. Anytime you come up with a project, make sure that the Ministry people are in the protocol, they are aware what you are trying to do from the beginning…You know, to become a friend of the government teams. Volunteer to participate in the technical working groups and make sure that they know you, they can trust you, you built that rapport.”*
-Modeler, Kenya

Transparent communication and data sharing between modelers and decision-makers is key to facilitating the use of modeled evidence. Having research institutes and knowledge brokers in the Ministry of Health with direct access to high level decision-makers helps to facilitate knowledge and data exchange in the Ministry. Software and databases that allow for the secure transmission of data are also critical for information sharing.

*“The fact that the Ministry has its own research centres, these are favorable factors, we meet our researchers every day, it means that we have access to their data that we can use. If we also want to seek expert advice, they are not very far away."*
-Decision-Maker, Burkina Faso
*“I think the biggest challenge has been the ‘black box’ issue. For groups that we work with that are open to sharing their code and reasoning behind the model structures up to the point about what assumptions they have made...that kind of transparency is great. I think the challenge has been when you have other modeling groups that are not being transparent, and they are feeding information straight to the policy makers in the absence of that transparency or critical review. Then you can end up with conflicting messages and you cannot tell when, where the problem is coming in, because it is a black box. That has been the main challenge, particularly at the start of the pandemic, before we were able to bring most of the groups together.”*
-Modeler, Kenya

Through mechanisms like task forces and committees that may be present at the federal and state department levels, decision-makers and research partners can come together to coordinate timelines, co-produce research questions, and engage in dialogue and debate about evidence that is produced to inform decision-making. These types of mechanisms and other government initiatives championing evidence-based decision-making in recent years have created platforms where new kinds of evidence, like modeled evidence, can thrive.

*“More and more decision-makers at the health sector level are more and more open to the use of data for decision-making. In reality, everyone wants the effectiveness of their projects and programs. So, they are looking for what has worked elsewhere, what has not worked well, also what has been found locally at the national level, so currently decision-makers are looking to know the success factors of their project."*
-Boundary Organization Rep, Burkina Faso
*“Earlier they were not bothered about the evidence. But nowadays without evidence, even if they cannot take any decision, they will be questioned. And because of the nature and kind of grilling that is going through, whether it is an academic or in administrators, evidence is definitely something which they cannot ignore and they have to generate.”*
-Knowledge Broker, India

***Environmental***

The lack of quality data and modeler access to data limits the production of models. Data are often collected by different agencies and can result in fragmented, program-specific outputs that are partial or biased and of limited value to modelers. Further, lack of communication between the modeling community and data gathering entities can lead to a misunderstanding about what is needed.

*“One thing I understand is that you can't blame the system for not collecting useful data because people who use the data like us haven't communicated that this is what we want. There is a difference between the system, the system that is capable to collect the data, and the system that is capable to analyse it. But they haven't sat across the table and said that this is what we want.”*
-Knowledge Broker, India
*“Ah, that data, yes, that data exists, poorly labelled. There is no dictionary in some of these data sets, the answers to some of the questions, like, for example, if we are using reporting tools, the reporting tools could be saying people are answering the questions in two different ways and that also affects the...and we also have to do a lot of, ‘What do they mean by this? What do they mean by that?’ and following up. So, the data that you are working with and [it] not being great is a reality.”*
-Modeler, Kenya

Some decision makers also expressed that they felt models that were built exclusively on foreign data (such as many of the models produced in the early COVID-19 pandemic) were of limited value for local decision-making.

*“What are you modeling? Think about it. We don’t know the number of persons that are being born in this country. We don’t know how many people are dying. We don’t know the age distribution. So, where will the modeling data come from?”*
-Decision-Maker, Nigeria

Sustained funding from internal government and external donor sources is needed to support researchers in generating modeled evidence in a well-established, routine, and sustainable manner. Funding is also needed to ensure intermediaries or boundary organizations are equipped with skills, spaces, and tools to facilitate knowledge exchange and to strengthen capacity for evidence-based decision-making in government through skill and awareness building.

*“The first question you asked me was how many people are working on this. I only have eight people and they are working part time because there are other duties that they have. The kind of long-term funding that organizations out there enjoy, like the London School, the Imperial College, is what enables them to also constantly be able to influence policy. I think that is a major shift. If you are funded based on a small project, then we don’t grow enough capacity like what you have heard now.”*
- Modeler, Kenya
*“And also, I think funding may not be enough to do high-quality research as much.”*
-Knowledge Broker, India
*“Well, practically, you know the way research is in Nigeria. It is the grant you get…that will determine whether you model or not.”*
-Modeler, Nigeria

The rapidly evolving nature of COVID-19 spurred widespread cooperation among countries in epidemiologic modeling to rapidly inform measures to curtail the spread of the virus and protect citizens. Informants noted how quickly decision-makers in ministries of health mobilized to create spaces and structures for bringing together decision-makers, modelers, epidemiologists, and other research partners, with clearly defined roles and responsibilities for each group.

*“There is no doubt COVID is definitely a bad thing. A whole lot of people died. But the silver lining of the cloud is that public health has been put at the forefront and the importance of investing in public health has been understood by a lot of people. So, people who were not heard so much of us right now are being heard. So, this is the right time to put forth and sensitize on the importance of the advantages of modeling data and things like health technical assistance and implementation.”*
-Knowledge Broker, India
*“Absolutely, because it was an opportunity for us to discuss with people who are not mathematicians, we exchanged with doctors, biologists and others, people who wanted to understand what we had done...And the model was used by the ministry and ourselves, it reassured us that what we do has applications, as long as we ourselves go to the decision-makers."*
-Modeler, Burkina Faso

### Mechanisms that enable exchange between modelers and decision-makers

We observed a range of mechanisms designed to facilitate exchange between modelers and researchers in the four study countries pointing to a shared recognition of the value in bringing diverse stakeholders together to advance evidence use in decision-making through institutional structures ( Table 5). Except for the dedicated task forces and committees that were mobilized quickly to respond to COVID-19, these mechanisms provide a space for debating evidence in the health sector broadly, with modeled outputs featuring as one type of evidence. There is an opportunity for country actors and partners to build on the momentum of the modeling-specific activities and structures introduced during COVID-19 – strengthening what has worked well and making improvements where needed.

**Table 5.** Mechanisms for enabling the translation of modeled evidence for decision-making.

	Government Advisory Groups	Consortium	Working Trainings	Formal Government Research Partnerships
**Definition**	Government-led advisory groups, task forces, or technical committees of experts & modelers that review available evidence & advise the government	Partnerships between NGOs, research/academic institutions & other stakeholders that regularly review & discuss evidence to provide guidance and advocacy to decision- makers	Training sessions, often organized by or with the government, that bring together researchers to develop modeling capacity through the collaborative development of a model	Formal ad hoc partnerships, including contractual arrangements & memoranda of understanding, established by the government with organizations that develop models to jointly explore key research questions
**Examples**	Nigeria’s National Council on Health	• India’s SARS-CoV-2 Genome Sequencing Consortium • Nigeria’s COVID-19 Research Coalition	• India’s Cochrane & Campbell Collaboration trainings • India’s Center for Global Development International Decision Support Initiative	Kenya MoH’s commission of a report on COVID modeling efforts
**Strengths**	• Allows for visibility of available evidence • Provides space for discussion & debate • Improves transparency • Directly tied to decision- makers	• Allows for wide visibility of available evidence • Provides space for discussion & debate • Improves transparency	• Develops capacity • Encourages transparency and collaboration • Promotes government leadership in modeling	• Intentional collaboration • Clear expectations
**Pitfalls**	May have limited membership	May not have direct ties to government	Requires organizational & convening capacity, including funding for experts	Certain partners may have favored status, limiting the pool of modeling expertise & diverse perspectives

In some fields (e.g., environmental science), stand-alone entities that are commonly referred to as boundary organizations and often independent of the research and policy sides, play a key role in facilitating knowledge exchange between research and policymaking communities. Knowledge brokers also play a role in facilitating exchange between research producers and users but typically are embedded within research / modeling organizations. We find that these roles apply to the modeling ecosystem as well. Specific mechanisms that aim to facilitate dialogue, debate, and communication in a boundary organization capacity include entities like the Nigeria National Council on Health. Mechanisms operating in a knowledge broker capacity include formal government research partnerships. Our research partners identified advisory groups as a common engagement mechanism for supporting modeling activities in all countries.

Finally, we observed in the small sample of four countries included in this study, that as capacity to produce, translate, and use modeled evidence develops, stand-alone boundary organizations tend to be replaced by knowledge brokers in modeling organizations.

## Conclusion

This study highlights the importance of taking an ecosystem approach to supporting modeling activities, considering different actors and how they interact to inform the production, translation, and use of modeled evidence. Structured interaction that promotes dialogue, debate, and joint sensemaking between the producers and users of evidence is critical to informing and influencing the use of evidence in decision-making. An ecosystem approach also means paying attention to the capabilities needed at all levels of the system – individual, organizational, and environmental to strengthen the use of evidence in decision-making.

Co-production approaches that facilitate iterative model development informed by the priorities of decision-makers, are a promising way to enhance decision-maker understanding of models, ensure relevance of models, and improve the likelihood that emerging recommendations will be considered. A collaborative engagement model like co-production that brings evidence producers and users together, can enhance decision-maker knowledge and skills in interpreting and using evidence. Researchers in turn can build a better understanding of the problems and issues that decision-makers need answered, become more attuned to policy windows, and foster a shared culture that promotes production and use of relevant evidence. Long-term funding that recognizes the complexity of these interactions and resources the different engagement mechanisms described in the paper is needed to build strong modeling ecosystems. Finally, lessons learned from the COVID-19 pandemic point to existing and functional researcher decision-maker engagement mechanisms and momentum in modeling activities that we hope the study countries and their partners will continue to build on.

## Recommendations

Modeled evidence is one source of input in a public health decision process that should be situated in the broader evidence system of countries. Strengthening the use of public health disease modeling in policy and planning involves an ecosystem of actors working in alignment to improve country-level evidence systems, including funders, modelers, boundary organizations or knowledge brokers, and decision-makers. Our recommendations are tailored to these groups ( Table 6– Table 9).

**Table 6.** Recommendations for modelers.

Recommendations	Why this is important
Invest in **building relationships with decision-makers, both** **formal and informal** to better understand research needs and emerging policy priorities	• Decision-makers are more likely to engage in discussion about research or modeled evidence with partners they trust • Collaboration during a crisis like COVID-19 is easier when positive working relationships between decision-makers and modelers already exist
Develop models that are **responsive to the priorities of** **decision-makers and the needs of public health organizations** **and communities**, and incorporate **local and regional data**	• The likelihood that decision-makers will use modeled outputs is higher when the model is relevant to decision needs • Decision-makers place higher trust in local data
Engage decision-makers **early and throughout the process of** **developing models**	• When decision-makers are consulted and engaged in the process of developing models, they develop a better understanding and awareness of the role that modeling can play in informing decisions • Continuous and iterative engagement can help to ensure the relevance of models • Co-production can help to build trust in the relationship between decision-makers and modelers
**Commit to communicating modeling assumptions and ** **outputs in clear and easy-to-understand formats** for use in decision-making	• Communication can help to improve the relevance of models • Decision-makers who trust and understand modeled outputs are more likely to use them to inform policy and practice

**Table 7.** Recommendations for Boundary Organizations and Knowledge Brokers.

Recommendations	Why this is important
Invest in **building awareness of and buy-in for the use of** **modeled evidence**	• When decision-makers understand the value of using modeled evidence, they are more likely to draw on it to inform policy and practice • At a basic level, decision-makers should understand when and why they should use modeled evidence, how to frame a research question, and how to use the evidence in informing policy or recommendations
Create **spaces to review and debate evidence, iterate on** **models, discuss implications for a decision process**	• These spaces are an opportunity to bring different voices to the table and ensure varying perspectives are heard in efforts to make sense of the evidence • When decision-makers and researchers to come together in a structured and routine way, the more likely their communication will improve – helping to increase decision-maker understanding of models and modeler awareness of decision-maker needs
**Guide and support modelers in communicating research** **outputs in clear and easy-to-understand formats** such as policy briefs, PowerPoint presentations, checklists, and fact sheets	• Decision-makers are not likely to make use of modeled evidence they do not understand

**Table 8.** Recommendations for Decision-makers.

Recommendations	Why this is important
Strengthen evidence systems, **including data** **accessibility and transparency**	• If the underlying data that are used to inform models are inaccurate, the evidence produced can be confusing to decision-makers and is less likely to be used
**Improve coordination with the modeling** **/ research community** through formal arrangements, technical working groups, or other structures	• Governments have convening power to bring different partners together from government, civil society, research and academic institutions, and funders. Build on the engagement mechanisms that worked well during COVID19 – improved coordination can facilitate routine sharing among different partners and improve the robustness of the evidence
**Build a culture of evidence use** by incentivizing evidence use – strengthen capacity and promote a culture of learning	• Decision-makers are more likely to use evidence, when the routinely engage with researchers and know how to find, appraise, and apply evidence • A culture of learning that promotes iterative modeling activities helps to ensure decision-maker and community needs are prioritized
**Increase funding** for public health disease modeling	• To improve the use of modeled evidence in decision-making sustained support for strengthening the capacity of modelers, knowledge brokers, and decision-makers is critical

**Table 9.** Recommendations for Funders.

Recommendation	Why this is important
Take an **ecosystem approach to** **investing in modeling**	• Different actors must be engaged to effectively move a model from the design phase, through creation, to eventual impact on policy. The evidence-to-decision- making ecosystem varies widely between countries – mapping the landscape and assessing its strengths and limitations is an important first step for an effective investment. • This approach is also useful for identifying existing capacity, including knowledge translation efforts – building on existing structures can help avoid duplication and ensure ownership and sustainability.
**Fund policy-engagement activities** **flexibly,** not just the production of models, as part of grantmaking	• Policymaking is relational – relationships are critical to ensuring models are relevant and decision-focused. It takes time to build relationships but often this aspect of policy work is not funded, which can signal that it is not valued. • Decision-making processes are often messy and unpredictable. Flexibility in grantmaking that also acknowledges what it takes to build relationships, would enable modelers to support critical decision windows as they arise.
**Center country research priorities** **and strengthen country evidence** **infrastructures**	• Strong data and research systems are needed to support overall use of evidence in government. Modeled evidence is one source of evidence in a decision process – when evidence systems are stronger, modeling activities are likely to be stronger too. • A focus on country-level research priorities and agendas will ensure relevance and help to strengthen data systems and other needed inputs.

## Data Availability

The audio transcripts and survey responses are not openly available for data protection reasons because, despite removing identifiable information such as names and organizational affiliations, we risk revealing individual identities through the highly specific and detailed interview responses. As part of the written consent agreement with participants, we assured them of anonymity when presenting synthesized findings. Further, in our IRB approval we agreed to the following condition, “Data files will be password protected and shared only between the field interviewers, field supervisors, and the research team.” The research team can provide additional anonymized quotes relevant to specific findings in the paper and access to select survey data on request. All requests must be made by email to Leah Ewald at lewald@r4d.org and should include a detailed rationale. Access to additional quotes will only be granted for legitimate research purposes. Figshare: “Understanding evidence ecosystems: What influences the production, translation, and use of modeled evidence in Burkina Faso, Nigeria, India, and Kenya?" https://doi.org/10.6084/m9.figshare.24216903.v2 ( Taddese *et al.*, 2023) This project contains the following extended data: Country summary reports Survey questionnaire and interview questions Data are available under the terms of the Creative Commons Attribution 4.0 International license (CC-BY 4.0).
