# Supplementary material for: Understanding evidence ecosystems: What influences the production, translation, and use of modeled evidence in Burkina Faso, Nigeria, India, and Kenya?
Source: Gates Open Res. 2024 Oct 24;7:135. Originally published 2023 Dec 13. [Version 2] doi: 10.12688/gatesopenres.14973.2 (PMC11333661; doi:10.12688/gatesopenres.14973.2)
Supplement: Peer review history

# Reviewer report: Reviewer response for version 2

**DOI:** 10.21956/gatesopenres.17695.r38668

**Published:** 2024-12-18

**Authors:** Milena Lopreite

**Affiliations:** 1 University of Calabria, Arcavacata, Italy

The theme is  interesting as well as the methodology, but I suggest to approve the paper   after a revision with some refinements as the following:

1. Introduction: Please motivate why the authors include in the analysis only the following countries: Burkina Faso, Nigeria, India, and Kenya.
2. Introduction: Please add the background of literature.
3. Introduction:Please specify better the contribution of the authors with respect to the existing literature.
4. Data collection: Have you tested the questionnaire before its administration?
5. Data collection: Please define better the inclusion and exclusion criteria of the interviewees.
6. Data analysis : Please move the limitations discussed in this section to the last part of the conclusion.
7. Data analysis: Could you discuss whether exists or not difference in the interviews’ reply between male and female?
8. Facilitators & inhibitors influencing production, translation, and use of modeled evidence: Please summarize the responses of the interviewees.
9. Please discuss better in the “conclusions” the policy implications of the study.
10. A linguistic review is strongly suggested.

Is the work clearly and accurately presented and does it cite the current literature?

Partly

If applicable, is the statistical analysis and its interpretation appropriate?

Partly

Are all the source data underlying the results available to ensure full reproducibility?

Partly

Is the study design appropriate and is the work technically sound?

Partly

Are the conclusions drawn adequately supported by the results?

Partly

Are sufficient details of methods and analysis provided to allow replication by others?

Partly

Reviewer Expertise:

NA

I confirm that I have read this submission and believe that I have an appropriate level of expertise to confirm that it is of an acceptable scientific standard, however I have significant reservations, as outlined above.

# Reviewer report: Reviewer response for version 2

**DOI:** 10.21956/gatesopenres.17695.r38334

**Published:** 2024-11-15

**Authors:** Zipporah Bukania

**Affiliations:** 1 Kenya Medical Research Institute, Nairobi, Kenya

Thank you, authors, for the revised paper, reads much better, I have however a few areas that are yet to be fully addressed.

 Study design; I note your improvements and indication that it was a sequential mixed method- I think this too needs to be specified. You need to further specify which  sequential mixed methods (exploratory or explanatory). Detail, which was done to inform the other, e.g. Was qual done to inform the focus of quant or otherwise.  However, I am also struggling to understand what  quantitative data was collected/used on the study.

 You do mention that: “ *The research teams conducted a quantitative analysis of survey results”  *which survey results are referred to here?  What are the variables? What is the sample size?  I do not see where you mention what quantitative data is available? Could you expound on this.  Which is the quantitative data? What quantitative method was used?  

 The desk review seems to have focused on identifying the key informants and the fact that there is data on the demographics of the informants does not qualify the data to mean quantitative. Its basically aa description of the participant.

 If quantitative data was not collected or retrieved from reviews, then you might not need to talk of mixed methods as a study design but rather, keep the study design to qualitative design.

 I recommend that you report your design as qualitative-methods. The details in this paper do not qualify it for mixed methods.

 Results: You have provided proportions of participants ( note in qualitative studies, it's recommended to use participants, while respondents are used for quantitative studies). Against what numbers are these proportions?  What were the total No of participants for which you have derived the proportions?

Is the work clearly and accurately presented and does it cite the current literature?

Yes

If applicable, is the statistical analysis and its interpretation appropriate?

Not applicable

Are all the source data underlying the results available to ensure full reproducibility?

Partly

Is the study design appropriate and is the work technically sound?

Partly

Are the conclusions drawn adequately supported by the results?

Partly

Are sufficient details of methods and analysis provided to allow replication by others?

No

Reviewer Expertise:

Public health, Nutrition, sufficient exposure on qualitative research methods

I confirm that I have read this submission and believe that I have an appropriate level of expertise to confirm that it is of an acceptable scientific standard, however I have significant reservations, as outlined above.

# Reviewer report: Reviewer response for version 2

**DOI:** 10.21956/gatesopenres.17695.r38332

**Published:** 2024-10-31

**Authors:** Justin Pulford

**Affiliations:** 1 Liverpool School of Tropical Medicine, Liverpool, UK

Recommend revisions have been adequately addressed

Is the work clearly and accurately presented and does it cite the current literature?

Yes

If applicable, is the statistical analysis and its interpretation appropriate?

Not applicable

Are all the source data underlying the results available to ensure full reproducibility?

Partly

Is the study design appropriate and is the work technically sound?

Partly

Are the conclusions drawn adequately supported by the results?

Yes

Are sufficient details of methods and analysis provided to allow replication by others?

Partly

Reviewer Expertise:

Research system strengthening

I confirm that I have read this submission and believe that I have an appropriate level of expertise to confirm that it is of an acceptable scientific standard.

# Reviewer report: Reviewer response for version 1

**DOI:** 10.21956/gatesopenres.16306.r37529

**Published:** 2024-08-19

**Authors:** Zipporah Bukania

**Affiliations:** 1 Kenya Medical Research Institute, Nairobi, Kenya

A good study focus and is quite relevant given the need for evidence for decision-making. However, the authors need to be very clear on which mixed methods design they have used. It's not enough to just mention mixed methods, but rather specify, eg. was sequential or concurrent, etc. The authors can review the different mixed methods designs and identify which one is the correct one. 

 The details under study design do not speak to study design. I do not think it necessary to mention the exclusion of South Africa at this point, in any case, their data is not included. That could be captured under limitations. 

 The study design section should majorly focus on design.

 I would expect the design section to give details on the focus areas of inquiry,  not just which organization participated in which activity.

 While qualitative approaches allow for convenient sampling, in Kenya, the selection of participants as described could be construed to indicate biases. I would think that the best approach was to select respondents who have roles /participation or work in evidence-based and modeling settings. 

 The authors should minimize the justification of why certain actions were not done. I think a clear coherent detailed mention at once will be sufficient. 

 The design could have been strengthened with other qualitative approaches like the workshop approach 

 Did the study have any limitations? 

 results:

 how was the distribution of the different professionals? how many modelers, researchers, etc.

 the results are good but I miss the triangulation of the desk review and the qualitative outputs.  what was in the desk review and how does it relate to interview findings?  what are the strengths of modeled evidence? 

 The conclusion is good but, could be improved by including where the main setback in the evidence and decision-making is? where should countries identify as challenges that can be addressed to improve these processes? for example is there adequate budget allocation to address the interaction and co-production? did the study note if there is sufficient Human Resources to support this?

Is the work clearly and accurately presented and does it cite the current literature?

Yes

If applicable, is the statistical analysis and its interpretation appropriate?

Not applicable

Are all the source data underlying the results available to ensure full reproducibility?

Partly

Is the study design appropriate and is the work technically sound?

Partly

Are the conclusions drawn adequately supported by the results?

Partly

Are sufficient details of methods and analysis provided to allow replication by others?

No

Reviewer Expertise:

Public health, Nutrition, sufficient exposure on qualitative research methods

I confirm that I have read this submission and believe that I have an appropriate level of expertise to confirm that it is of an acceptable scientific standard, however I have significant reservations, as outlined above.

# Response

**Published:** 2024-10-17

**Authors:** Abeba Taddese

**Affiliations:** Results for Development Institute, Washington, District of Columbia, USA

Dear Reviewer,

 Thank you for taking the time to review our article and offer your valuable feedback. (please find Responses in bold)

 The details under study design do not speak to study design. I do not think it necessary to mention the exclusion of South Africa at this point, in any case, their data is not included. That could be captured under limitations.  

**We have kept mention of South Africa in the study design because this is where we mention the group of countries we started with and want to establish for the reader early why they might not see mention of South Africa again for a few pages, but we have also added a sentence under limitations.**

 The study design section should majorly focus on design. 

**We have added some additional details.**

 I would expect the design section to give details on the focus areas of inquiry,  not just which organization participated in which activity. 

**Thank you for this feedback.**

 While qualitative approaches allow for convenient sampling, in Kenya, the selection of participants as described could be construed to indicate biases. I would think that the best approach was to select respondents who have roles /participation or work in evidence-based and modeling settings.  

**Thank you for this feedback.**

 The authors should minimize the justification of why certain actions were not done. I think a clear coherent detailed mention at once will be sufficient.  

**Please see above explanation for our mention of South Africa in a few different places.**

 The design could have been strengthened with other qualitative approaches like the workshop approach  

**Thank you for this feedback.**

 Did the study have any limitations?  

 results: 

 how was the distribution of the different professionals? how many modelers, researchers, etc. 

**This information is included in Table 1.**

 the results are good but I miss the triangulation of the desk review and the qualitative outputs.  what was in the desk review and how does it relate to interview findings?  what are the strengths of modeled evidence?  

**The desk review was used to map the primary players in the modeling ecosystem of the study countries. These details are included in the country reports included in the extended data section. We did not assess modeled evidence – our focus on understanding the mechanisms that facilitate use of modeled evidence**

****

 The conclusion is good but, could be improved by including where the main setback in the evidence and decision-making is? where should countries identify as challenges that can be addressed to improve these processes? for example is there adequate budget allocation to address the interaction and co-production? did the study note if there is sufficient Human Resources to support this? 

**We offer some suggestions in the recommendations section.**

# Reviewer report: Reviewer response for version 1

**DOI:** 10.21956/gatesopenres.16306.r37525

**Published:** 2024-08-13

**Authors:** Justin Pulford

**Affiliations:** 1 Liverpool School of Tropical Medicine, Liverpool, UK

I found this to be a well-conceived and novel study that offers pragmatic recommendations for improving the impact of modelled evidence within a global health context. I have a small number of recommendations, all of which I would consider minor:

 The authors state a focus on ‘knowledge brokers’ and ‘boundary organisations’ in the last paragraph of the introduction, but do not go on to define what these terms mean until later in the manuscript.  I recommend including a note here in brackets that definitions for these terms are presented in Table 2.

 More detail is needed in the data analysis sub-section.  The authors state they conducted a quantitative analysis of survey data, although no details of this analysis are provided.  There is also very little quantitative data presented in the results section which raises further questions as to what analyses were conducted or how the quantitative data have been reported. 

 More detail is also needed re how the qualitative data were prepared and analyzed. Were interviews transcribed and translated?  Was the coding completed manually or with the assistance of software.  Did the two coders from each country code independently and, if so, how were disagreements resolved etc.

 Figure 1.  The heading for the second column ‘Ecosystem classification…’ appears incomplete.

 Are the terms ‘universities’ and ‘academic institutions’ being used interchangeably or are they intended to have different meanings?  If the former, I suggest using a consistent term.  If the latter, define.

 It is not always entirely clear what data sources are informing statements presented in the result section.  There is often reference to key informant interview data, but less reference to survey data and no ‘precise’ statements that you often associate with quantitative analysis (e.g. 55% of…).  Are the survey and interview data being presented separately or are common trends across both being reported?

Is the work clearly and accurately presented and does it cite the current literature?

Yes

If applicable, is the statistical analysis and its interpretation appropriate?

Not applicable

Are all the source data underlying the results available to ensure full reproducibility?

Partly

Is the study design appropriate and is the work technically sound?

Partly

Are the conclusions drawn adequately supported by the results?

Yes

Are sufficient details of methods and analysis provided to allow replication by others?

Partly

Reviewer Expertise:

Research system strengthening

I confirm that I have read this submission and believe that I have an appropriate level of expertise to confirm that it is of an acceptable scientific standard, however I have significant reservations, as outlined above.

# Response

**Published:** 2024-10-17

**Authors:** Abeba Taddese

**Affiliations:** Results for Development Institute, Washington, District of Columbia, USA

Dear reviewer,

 Thank you for taking the time to read our article and offer your valuable feedback.

 The authors state a focus on ‘knowledge brokers’ and ‘boundary organisations’ in the last paragraph of the introduction, but do not go on to define what these terms mean until later in the manuscript.  I recommend including a note here in brackets that definitions for these terms are presented in Table 2. 

**This has been added.**

 More detail is needed in the data analysis sub-section.  The authors state they conducted a quantitative analysis of survey data, although no details of this analysis are provided.  There is also very little quantitative data presented in the results section which raises further questions as to what analyses were conducted or how the quantitative data have been reported.  

**We have added some additional details – detailed country-level analysis can be found in the country reports included in the extended data section.**

 More detail is also needed re how the qualitative data were prepared and analyzed. Were interviews transcribed and translated?  Was the coding completed manually or with the assistance of software.  Did the two coders from each country code independently and, if so, how were disagreements resolved etc. 

**Interviews were transcribed and translated coding was completed with the assistance of software of choice which we have added to the paper.** **Coders coded the first two interviews together and met to discuss and resolve differences in coding. We did not test the inter-rater reliability of codes after the initial two interviews.**

 Figure 1.  The heading for the second column ‘Ecosystem classification…’ appears incomplete. 

**This figure has been updated.**

 Are the terms ‘universities’ and ‘academic institutions’ being used interchangeably or are they intended to have different meanings?  If the former, I suggest using a consistent term.  If the latter, define. 

**We have corrected instances in which the terms were used interchangeably and added a definition clarifying how we've used the terms. Use of academic includes institutes and colleges.**

# Reviewer report: Reviewer response for version 1

**DOI:** 10.21956/gatesopenres.16306.r35715

**Published:** 2024-02-27

**Authors:** So Yoon Sim

**Affiliations:** 1 World Health Organization, Geneva, Switzerland

Thanks for this important study which examined evidence ecosystems and identified multiple levels of factors influencing production, translation, and use of modeled evidence.

 The study generated rich insights that could inform policy and agenda setting related to translation of modeled evidence. Furthermore, recommendations from this study could guide diverse actors to assess strengths and weaknesses of each translation mechanism and develop action plans to enhance evidence-informed decision making.

 Please see my minor comments below regarding methodology:

 Could you please provide details about the inclusion criteria for seven countries of strategic importance to BMGF? Which countries are these? How were they selected by BMGF?

 Similarly for the ecosystem classification ("nonexistent," "nascent," "optimizing," and "flourishing"), could you please explain how each category is defined? E.g. What are some requirements for countries to be classified into "flourishing"?

 Was inter-rater reliability of the code was tested? Could you please provide details on degree of agreement between research teams?

 Table 2: how consistent are these definitions with those used in the current literature? Sources?

 Could the codebook developed by R4D be made available in the appendix?

Is the work clearly and accurately presented and does it cite the current literature?

Yes

If applicable, is the statistical analysis and its interpretation appropriate?

Not applicable

Are all the source data underlying the results available to ensure full reproducibility?

No source data required

Is the study design appropriate and is the work technically sound?

Partly

Are the conclusions drawn adequately supported by the results?

Yes

Are sufficient details of methods and analysis provided to allow replication by others?

Partly

Reviewer Expertise:

health economics, vaccine impact modeling, knowledge translation

I confirm that I have read this submission and believe that I have an appropriate level of expertise to confirm that it is of an acceptable scientific standard.

# Response

**Published:** 2024-10-17

**Authors:** Abeba Taddese

**Affiliations:** Results for Development Institute, Washington, District of Columbia, USA

Dear reviewer,

 Thank you for taking the time to review our article and offer your valuable feedback.

 Could you please provide details about the inclusion criteria for seven countries of strategic importance to BMGF? Which countries are these? How were they selected by BMGF? 

 Response: We have clarified that these seven countries are those listed in Figure 1 and were offered as the starting group for the research because they are either BMGF focus countries or for known modeling activities.  

 Similarly for the ecosystem classification ("nonexistent," "nascent," "optimizing," and "flourishing"), could you please explain how each category is defined? E.g. What are some requirements for countries to be classified into "flourishing"?  

 Response: We have removed reference to the scale as it was a preliminary ranking based on the initial desk review and we do not include the results in this paper, instead we present a more comprehensive analysis based on our KII and survey findings. We have clarified that the desk review was used to estimate the level of activity and experience that each country had around modeling and the number and variety of actors engaged in modeling to allow us to select countries at a variety of stages in the development of their modeling ecosystem. 

 Was inter-rater reliability of the code was tested? Could you please provide details on degree of agreement between research teams?  

 Response: Coders coded the first two interviews together and met to discuss and resolve differences in coding. We did not test the inter-rater reliability of codes after the initial two interviews. 

 Table 2: how consistent are these definitions with those used in the current literature? Sources? 

 Response: We have added sources for the terms that are often defined differently in the literature. To our understanding the remaining definitions are consistent with current literature. 

 Could the codebook developed by R4D be made available in the appendix? 

 Response: Yes, we have added it.
